# The Application of the Logistic Equation Model to Predict the Remineralization Characteristics of Desensitizing Paste

**DOI:** 10.1155/2019/7528154

**Published:** 2019-10-03

**Authors:** Stanley Chibuzor Onwubu, Phumlane Selby Mdluli, Shenuka Singh, Obiora Cornelius Collins

**Affiliations:** ^1^Dental Sciences, Durban University of Technology (DUT), Durban, South Africa; ^2^Chemistry, Durban University of Technology (DUT), Durban, South Africa; ^3^Discipline of Dentistry, University of KwaZulu-Natal (UKZN), Durban, South Africa; ^4^Institute of Systems Science, Durban University of Technology, Durban 4000, South Africa

## Abstract

**Objectives:**

A mathematical model making using of the Verhulst logistic equation was developed to predict the remineralization behaviors of desensitizing paste.

**Methods:**

The input parameter used for the model was obtained experimentally by brushing twenty-one simulated dentin specimens for seven days with three sample groups, namely, EB@TiO_2_, Colgate Pro-relief, and Sensodyne repair (*n* = 7). A field emission scanning electron microscope (FESEM) and ImageJ software were used to observe and measure the % occluded ratio of the dentin surface. The model fittings for the three sample groups were carried out using the built-in MATLAB least-squares fitting routine fmincon in the optimization toolbox.

**Results:**

The results suggest that the experimental parameter were in agreement with the model. It was found that the logistic equation model can make a future prediction of the remineralization pattern for EB@TiO_2_ and Colgate Pro-relief. It was, however, found that the trajectory for the Sensodyne repair was a bit complex, thus making the prediction difficult.

**Conclusions:**

Overall, the salient feature of this study suggests that the logistic equation could be used to predict the remineralization behavior of desensitizing paste in the management of sensitive tooth.

## 1. Introduction

Over the last decade, dentin hypersensitivity (DH) has been extensively researched owing to its widespread prevalence and noticeable painful oral health problem affecting many individuals [1]. A previous study [2] reported that more than 80% of children and up to 43% of adult's population suffer from dental pain associated with DH. More worrisome is that DH negatively affects the quality of life of dental patients if left untreated [3]. Consequently, numerous dentin remineralization strategies have been proposed in the literature [4, 5] for the management of DH. Amongst these, the use of biomaterials such as bioactive glass and proargin has been reported to effectively occlude the open dentinal tubules [6]. While bioactive glass, for example, is noted to provide some substantial relief to patients [6], the overall duration of the treatment strategy using this material remain elusive in both saliva and without saliva.

Although bioactive glass occludes patent dentinal tubules by supplying calcium (Ca^2+^) and phosphate (PO_4_^3−^) ions in an optimum oral environment to form hydroxycarbonate apatite (HCA) [7, 8], in some patients, however, particularly those with conditions of hyposalivation and xerostomia, the flow of saliva is limited [9]. As reported in the literature [10, 11], saliva facilitates the deposition of the trap Ca^2+^ and PO_4_^3−^ ions into open dentine tubules that gradually bring about tubule sealing or occlusion. Hence, it is sufficient to assume that the effectiveness of the bioactive glass will be less effective in patients with a limited flow of saliva.

In an attempt to address the aforementioned concerns of limited salivary flow, proargin technology was developed by Kleinberg in 2002 based on the role saliva plays in naturally occluding dentinal tubules [6]. According to [6], proargin comprises arginine (an amino acid with pH 6.5–7.5), bicarbonate, pH buffer, and calcium carbonate. It is reported by Hamlin et al. [12] that the interaction of arginine and calcium carbonate at physiological pH subsequently attracts a calcium-rich layer that binds to the negatively charged dentin surface. This, in turn, facilitates the infiltration of calcium resulting in the blocking of the dentinal tubules [13]. However, Yang et al. [14] found that Colgate Pro-relief showed no significant changes after treatment and immersion in artificial saliva for 14 days.

Given the differences in the occluding characteristics for the aforementioned biomaterials in saliva and without saliva, a new biomaterial from eggshell waste and titanium dioxide (EB@TiO_2_) is proposed as an alternative occluding material for DH management. Whilst a recent study has demonstrated the occluding characteristics of EB@TiO_2_ [15], the time required to completely and effectively occlude the dentine tubules in a simulated oral environment is yet to be established. Equally essential, and in line with the assertion of Schmidlin and Sahrmann [16], there is yet to be established a gold standard for DH management with a predictable and long-lasting relief of DH.

Importantly, mathematical modeling offers a different research perspective by overcoming some of the problems frequently encountered in an experimental study [17]. Essentially, using numeral tools, Ilie et al. [17] assumed that it is possible to fashion out a controlled environment to address the challenges of long time period needed to effectively study the biological process. In the last decade, a different mathematical model has been proposed by various scholars to investigate dental hard tissues, most nevertheless, focus largely on dental caries, and tooth demineralization process [17–19]. Despite the numerous models developed to study dental tissues, there is limited evidence that suggests the use of a mathematical model to predict the remineralization potentials of desensitizing agents on dentin tubules. This study uses the logistic equation model as a tool for the prediction of the effectiveness of desensitizing agents in occluding dentin tubules.

### 1.1. Logistic Equation

The logistic equation was first proposed by the seminal work of Pierre-Francois Verhulst (1844-1845). Verhulst derived the logistic equation to describe the self-limiting growth of biological population [20]. Interestingly, Sweilam et al. [21] assert that the logistic equation is described by a first-order ordinary differential equation. Their report resonates further with Murphy et al. [22] who noted that the logistic equation is formalized by the differential equation. Accordingly, it noted that the logistic model describes the growth of a population is limited by a carrying capacity of *b* [22]. Hence, the logistic equation assumes that the growth rate decreases linearly with size until it equals zero at the carrying capacity [22].

Ever since the discovery, the logistic equation has been extensively used in many scientific fields such as ecology, chemistry, population dynamism, mathematical psychology, political science, geoscience, statistics, economics, and sociology [23–26]. In ecology, for example, the logistic equation has been widely used to model the population growth where the rate of reproduction is proportional to both existing population and the amount of resources available [21]. This is expressed mathematically as follows:

(1) $$\begin{array}{c} \frac{dP}{dt}=rP\left(1-\frac{P}{K}\right), \end{array}$$

where *P* represents the population size, *r* is the constant that defines the growth rate, *K* is the carrying capacity, and *t* represents the time.

Another typical application of the logistic equation is in medicine, where the logistic differential equation is used to model the growth of tumors or to study the reaction of pharmacokinetics [20]. Here, the application of the logistic equation can be considered an extension of the abovementioned use in the framework of ecology, where *d*(*t*) is the size of the tumor at time *t* [21]. Given the predictive power of the logistic equation, this study aimed to develop a mathematical model (logistic equation model) to study the remineralization capabilities of three desensitizing paste, namely, EB@TiO_2_, proargin, and bioactive glass (NovaMin), in saliva and without saliva.

## 2. Materials and Methods

Food grade anatase titanium dioxide (CAS No: 13463677) was purchased from Sigma-Aldrich (Germany). Citric acid monohydrate was supplied by Merck (South Africa). Two different brands of toothpaste, namely, Sensodyne repair (GlaxoSmithKline, UK) and Colgate Pro-relief (Colgate-Palmolive, Poland), were bought from a popular shopping mall located at Durban (South Africa).

### 2.1. Preparation of Eggshell-Titanium Dioxide Composite (EB@TiO_2_)

Eggshell and titanium dioxide composite was prepared in accordance with the method reported in the literature [15]. 20 g of the fine eggshell powder was modified by adding 5 g of anatase titanium dioxide (≤15 *μ*m). The mixture was subsequently ball-milled for 200 min to obtain the eggshell-titanium dioxide composite (EB@TiO_2_).

### 2.2. Experimental Input Parameter

The experimental parameter was obtained from the remineralization test conducted in our laboratory. Twenty-one dentin specimens measuring 5 mm × 5 mm × 1 mm were prepared by sectioning perpendicular to the long axis of the teeth below the enamel-dentinal junction using a low-speed diamond saw under water cooling conditions. A sensitive model was simulated by soaking the specimens in 4% wt citric acid solution for 2 min. The specimens were then randomly assigned into three groups, namely, EB@TiO_2_, Colgate Pro-relief, and Sensodyne repair (*n* = 7).

Each specimen from the respective groups was brushed twice daily (morning and evening) for seven days with a toothbrush powered with 1.5 v alkaline battery (Oralwise, China) for 1 min and allowed to dry for 30 s before rinsing with deionized water. Brushing was performed at room temperature using 100 mg of each respective toothpaste. The slurry of EB@TiO_2_ was prepared by mixing 100 mg of the powder with 200 *μ*L of deionized water. After each brushing protocol, the specimens were immersed in saliva or without saliva. Using a field emission scanning electron microscope (FESEM; Carl Zeiss) operating at controlled atmospheric conditions at 20 kV, we examined the surface of the dentin after each day of brushing. The ratios of occluded tubules were computed using ImageJ software (National Institute of Health, USA, http://imagej.nih.gov./ij). This was calculated by dividing the area of occluded tubules by the total tubules area using ×1500 magnification images (*n* = 7). The mean values of the occluded area ratio were evaluated with 1-way analysis of variance (ANOVA). This was followed by a multicomparison test with Bonferroni correction (*α* = 0.05).

### 2.3. Model Description

The mathematical model considers that the size of dentin tubules (*S*) and the amount of calcium and phosphate deposits (*A*) influence significantly the time (*t*) needed to completely and effectively occlude the dentin tubules.

#### 2.3.1. Logistic Model

The following logistic equation model was proposed:

(2) $$\begin{array}{c} \frac{dX}{dt}=r_{x}X\;\left(1-\frac{X}{K_{x}}\right), \end{array}$$

where *X* is the percentage of tubules occluded, *r*_*x*_ is the rate at which *X* is being occluded, *K*_*x*_ is the maximum value of *X*, and *t* is the time to complete remineralization of dentin tubules. Unless otherwise stated, we will take *K*_*x*_=100% since it is the maximum value of *X*.

#### 2.3.2. Analytical Solution to the Model

By using the method of solving the first-order ordinary differential equation (separation of variable method), we obtain the analytical solution of the model equation (3):

(3) $$\begin{array}{c} X\left(t\right)=\frac{K_{x}\;C\;e^{r_{x}t}}{1\;+\;C\;e^{r_{x}t}}, \end{array}$$

where  *C*= *X*(0)/*K*_*x*_  − *X*(0) and *X*(0) is *X*(*t*) at *t* = 0 (i.e., initial value of *X*). Further analyses on model (2) show that the model has two equilibrium points: the trivial equilibrium point *X*^0^=0 and the positive equilibrium point *X*^*∗*^=*K*_*x*_. Conducting stability analysis about the two equilibrium states shows that the positive equilibrium point *X*^*∗*^=*K*_*x*_ is globally stable. This is easily established as *X*(*t*)⟶*X*^*∗*^=*K*_*x*_ as *t*⟶*∞*. This result shows that it is possible for *X*(*t*) to increase to the carrying capacity *K*_*x*_. On the other hand, the trivial equilibrium point *X*^0^=0 is unstable. This shows that it will be difficult for *X*(*t*) to decrease to zero.

#### 2.3.3. Model Fitting and Parameter Estimation

A model fitting and parameter estimation were conducted using our model to fit real data for the three samples (EB@TiO_2_, Colgate Pro-relief, and Sensodyne repair) for the two cases: with saliva and without saliva. The aim of these analyses is to show that the model we considered can be used to study as well as make future predictions on these samples. For the model fitting, we take the carrying capacity (*K*_*x*_) as 100% while the growth rate is estimated from the model fittings for all the samples. The model fittings were carried out using the built-in MATLAB least-squares fitting routine fmincon in the optimization toolbox.

## 3. Results

### 3.1. Experimental Parameter

Table 1 describes the % occluded area ratios of the dentin specimens brushed in seven days with or without artificial saliva. In the EB@TiO_2_-treated group, the % occluded area ratios observed for the specimens with saliva were significantly higher than those without saliva for day 2, 3, 4, 6, and 7 (*P* < 0.05). No differences were observed between the two groups in day 5 (*P* > 0.05). On the other hand, the group without saliva was higher than those with saliva on day 1 (*P* < 0.05).

For the Sensodyne repair group, the % occluded area ratios observed in days 1–7 for the group with saliva were statistically significantly higher than those observed without saliva (*P* < 0.05). In contrast, and for the Colgate Pro-relief group, the specimens brushed without saliva were consistently and significantly higher when compared against the group with saliva in each respective day (*P* < 0.05).

### 3.2. Predictability of the Model

**Figure 1:**
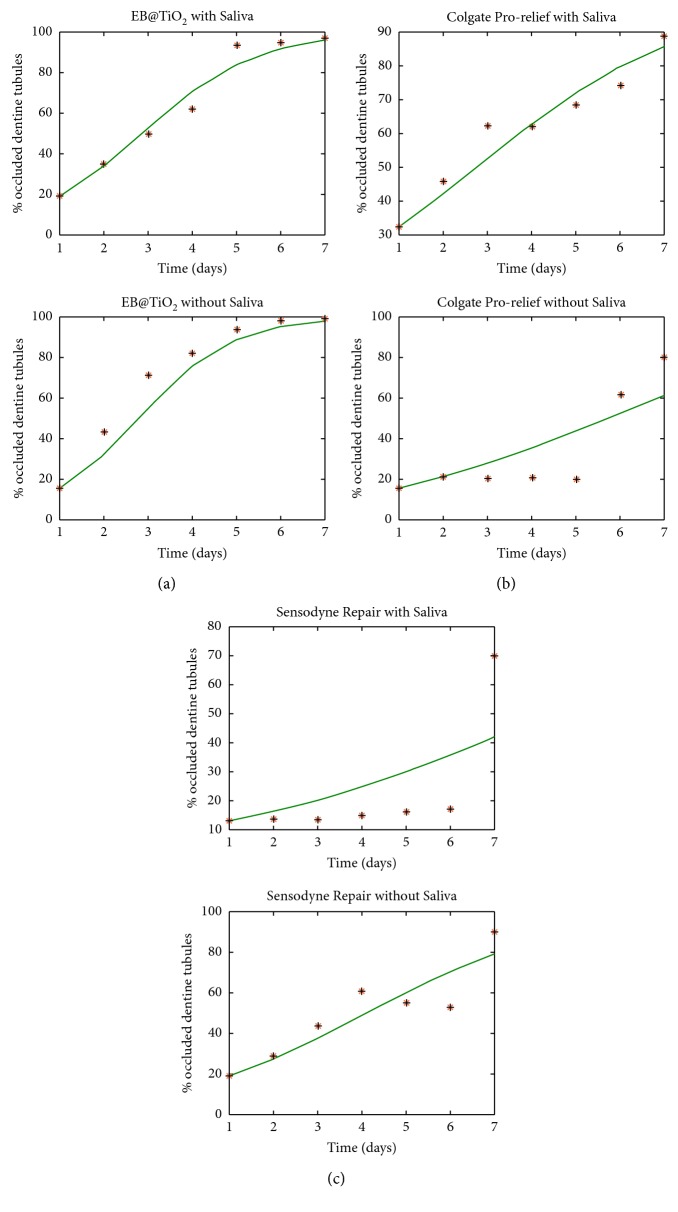
Model fitting of the desensitizing paste in saliva and without saliva.

The results of the model fitting for the three samples are presented in Figure 1. From the Figure 1, it can be observed that the proposed model produces a good fitting for two samples: EB@TiO_2_ and Colgate Pro-relief (for the two cases: with and without saliva), but did not produce a very good fitting for the Sensodyne repair.

The estimated parameters for the tubule occlusion rate (*r*_*x*_) is given in Table 2. For the specimens treated with EB@TiO_2_, it was observed that the *r*_*x*_ was higher (0.9500) with saliva when compared with the rate measured without saliva (0.7762). Similarly, the *r*_*x*_ measured for Sensodyne repair was higher in the specimens treated with saliva (0.4635) when compared against those treated without saliva (0.2646). In contrast, it was found that the *r*_*x*_ for specimens treated with Colgate Pro-relief without saliva was higher than those measured with the specimens treated with saliva.

## 4. Discussion

The purpose of this study was to investigate the use of the logistic equation model to predict the remineralization characteristics of desensitizing paste. Ilie [19] had advised that the parameters needed for developing a mathematical predictive model should be determined experimentally. In line with the author's advice, the input parameters used for the logistic equation model were determined through the remineralization test conducted on prepared bovine specimens. The findings suggest that the experimental results were in agreement with our model prediction.

Overall, the remineralization characteristics measured for EB@TiO_2_ were significantly better than those observed for Colgate Pro-relief and Sensodyne repair (*P* < 0.05). It was found that the remineralization behavior of EB@TiO_2_ in saliva and without saliva appear to follow the same pattern. More importantly, the model produces a good fitting for the dentin specimens treated with or without saliva (Figure 1). Predictably, and regardless of the salivary condition, it is assumed that EB@TiO_2_ would achieve complete occlusion of the dentin tubules at the end of seven days brushing. The remineralization ability observed for samples treated with EB@TiO_2_ may be attributed to the modification of the carbonate structure content in eggshell with titanium dioxide. This is in agreement with the suggestion of Cutler [27] that nanosized titanium dioxide together with abrasive materials promotes the occluding of dentin tubules. Another reason for the effective remineralization seen in the EB@TiO_2_ sample group could be attributed to the nanosized particle distribution in EB@TiO_2_ [28]. According to Nakashima et al. [29], nanosized calcium carbonate materials have unique high surface energy, thus facilitating the attachment of calcium-rich ions on the oral tooth surface.

With regard to the Colgate Pro-Argin paste, the model fitting suggests that the behavior with and without saliva differs. It was found that the model produced a better fitting for the samples treated without saliva (Figure 1). In addition, it is predictable that Colgate Pro-Argin would achieve a fast and complete occlusion of the dentin tubules after seven days of treatment. However, the model pattern for the samples treated with saliva immersion suggests that the effectiveness of proargin would require a longer period than seven days to effectively occlude the tubules. This is in agreement with the recent clinical finding that proargin achieved significant relief of DH after application for 24 weeks [3].

In terms of the Sensodyne repair, we could not achieve a good model fit to make an accurate prediction of the treatment. This may, however, be related to the quality of the input parameters [19]. Equally important, it was worth mentioning that the calcium (Ca^2+^) and phosphate (PO_4_^3−^) ions in NovaMin are protected by glass particles and thus would need to be trapped for it to effectively penetrate the dentin tubules [30]. The absence of saliva in the treated samples may have contributed to the complex trajectory behavior observed. In saliva, it is assumed that the trapped calcium and phosphate ions may be delayed in the release which could also have accounted for the complex trajectory observed for the samples treated with saliva immersion.

## 5. Conclusion

In this paper, we have demonstrated that our model can be used to study and make a future prediction for the two samples: EB@TiO_2_ and Colgate Pro-relief (for both cases: with and without saliva). For the Sensodyne repair, we discover from the figure that the trajectory for its real data is a bit complex. This makes it difficult to obtain a good fitting using our model. We hope to consider a more complex model that can fit the real data for Sensodyne repair in our future work.

## Figures and Tables

**Table 1:** Mean and standard deviation for the area ratios of the occluded tubules brushed in seven days with and without saliva.

	Day 1		Day 2		Day 3		Day 4		Day 5		Day 6		Day 7	
	WS	S	WS	S	WS	S	WS	S	WS	S	WS	S	WS	S
EB@TiO_2_	19 ± 2.6^a^	15.6 ± 2.2^b^	35.1 ± 4.4^a^	43.6 ± 4.7^b^	50.4 ± 4.3^a^	71.4 ± 2.8^b^	62.0 ± 3.7^a^	82.0 ± 3.1^b^	93.3 ± 2.3^a^	93.9 ± 3.0^a^	95.1 ± 1.6^a^	98.4 ± 1.9^b^	97.9 ± 1.3^a^	99.3 ± 1.0^b^
Colgate Pro-relief	32.3 ± 3.0^a^	16 ± 3.6^b^	45.7 ± 6.8^a^	21.4 ± 2.2^b^	62.3 ± 6.7^a^	20.3 ± 2.7^b^	62.0 ± 6.7^a^	20.9 ± 2.9^b^	68.6 ± 6.1^a^	19.9 ± 3.4^b^	74.3 ± 6.8^a^	61.9 ± 4.8^b^	88.9 ± 3.2^a^	80.1 ± 8.6^b^
Sensodyne repair	12.9 ± 3.6^a^	19.3 ± 3.5^b^	13.6 ± 2.5^a^	29.0 ± 2.2^b^	13.4 ± 2.8^a^	44.1 ± 4.9^b^	15.0 ± 3.3^a^	60.9 ± 4.6^b^	16.0 ± 3.1^a^	55.3 ± 5.3^b^	17.0 ± 3.7^a^	53.3 ± 6.2^b^	70.1 ± 4.2^a^	90.4 ± 3.0^b^

Values are mean ± standard deviations (*n* = 7). Different superscript letters indicate significant differences (*P* < 0.05). WS = without saliva; S = with saliva.

**Table 2:** Estimation of model parameters.

Parameters (*r*_*x*_)	Estimated by model fitting	
	Without saliva	With saliva
EB@TiO_2_	0.7762	0.9500
Colgate Pro-relief (proargin technology)	0.4230	0.3558
Sensodyne repair (NovaMin)	0.2646	0.4635

## Data Availability

The data used to support the findings of this study are included within the article.
